# Acute canagliflozin treatment protects against in vivo myocardial ischemia–reperfusion injury in non-diabetic male rats and enhances endothelium-dependent vasorelaxation

**DOI:** 10.1186/s12967-019-1881-8

**Published:** 2019-04-16

**Authors:** Alex Ali Sayour, Sevil Korkmaz-Icöz, Sivakkanan Loganathan, Mihály Ruppert, Viktor Nabil Sayour, Attila Oláh, Kálmán Benke, Maik Brune, Rita Benkő, Eszter Mária Horváth, Matthias Karck, Béla Merkely, Tamás Radovits, Gábor Szabó

**Affiliations:** 1 Department of Cardiac Surgery, Heidelberg University Hospital, Heidelberg, Germany; 2 Experimental Research Laboratory, Heart and Vascular Center, Semmelweis University, Városmajor u. 68, Budapest, 1122 Hungary; 3 Department of Pharmacology and Pharmacotherapy, Semmelweis University, Budapest, Hungary; 4 Department of Medicine I and Clinical Chemistry, Heidelberg University Hospital, Heidelberg, Germany; 5 Department of Physiology, Semmelweis University, Budapest, Hungary

**Keywords:** Sodium–glucose cotransporter-2 inhibitor, Canagliflozin, Myocardial ischemia–reperfusion injury, Cardioprotection

## Abstract

**Background:**

The sodium–glucose cotransporter-2 (SGLT2) inhibitor canagliflozin has been shown to reduce major cardiovascular events in type 2 diabetic patients, with a pronounced decrease in hospitalization for heart failure (HF) especially in those with HF at baseline. These might indicate a potent direct cardioprotective effect, which is currently incompletely understood. We sought to characterize the cardiovascular effects of acute canagliflozin treatment in healthy and infarcted rat hearts.

**Methods:**

Non-diabetic male rats were subjected to sham operation or coronary artery occlusion for 30 min, followed by 120 min reperfusion in vivo. Vehicle or canagliflozin (3 µg/kg bodyweight) was administered as an intravenous bolus 5 min after the onset of ischemia. Rats underwent either infarct size determination with serum troponin-T measurement, or functional assessment using left ventricular (LV) pressure–volume analysis. Protein, mRNA expressions, and 4-hydroxynonenal (HNE) content of myocardial samples from sham-operated and infarcted rats were investigated. In vitro organ bath experiments with aortic rings from healthy rats were performed to characterize a possible effect of canagliflozin on vascular function.

**Results:**

Acute treatment with canagliflozin significantly reduced myocardial infarct size compared to vehicle (42.5 ± 2.9% vs. 59.3 ± 4.2%, P = 0.006), as well as serum troponin-T levels. Canagliflozin therapy alleviated LV systolic and diastolic dysfunction following myocardial ischemia–reperfusion injury (IRI), and preserved LV mechanoenergetics. Western blot analysis revealed an increased phosphorylation of adenosine monophosphate-activated protein kinase (AMPK) and endothelial nitric-oxide synthase (eNOS), which were not disease-specific effects. Canagliflozin elevated the phosphorylation of Akt only in infarcted hearts. Furthermore, canagliflozin reduced the expression of apoptotic markers (Bax/Bcl-2 ratio) and that of genes related to myocardial nitro-oxidative stress. In addition, treated hearts showed significantly lower HNE positivity. Organ bath experiments with aortic rings revealed that preincubation with canagliflozin significantly enhanced endothelium-dependent vasodilation in vitro, which might explain the slight LV afterload reducing effect of canagliflozin in healthy rats in vivo.

**Conclusions:**

Acute intravenous administration of canagliflozin after the onset of ischemia protects against myocardial IRI. The medication enhances endothelium dependent vasodilation independently of antidiabetic action. These findings might further contribute to our understanding of the cardiovascular protective effects of canagliflozin reported in clinical trials.

## Background

Sodium–glucose cotransporter-2 (SGLT2) inhibitors are novel antidiabetic agents prescribed for a growing number of patients with type 2 diabetes mellitus (T2DM) worldwide. Recently, the landmark EMPA-REG OUTCOME clinical trial [1] reported that the SGLT2 inhibitor empagliflozin, when added to standard care, significantly reduced the risk of major cardiovascular events (the composite of death from cardiovascular causes, non-fatal myocardial infarction and non-fatal stroke) compared to placebo (hazard ratio [HR], 0.86; 95% confidence interval [CI] 0.74–0.99; P = 0.04). The CANVAS Program [2] reported a similar effect on the composite outcome with the SGLT2 inhibitor canagliflozin in T2DM patients with high cardiovascular risk (HR, 0.86; 95% CI 0.75–0.97; P = 0.02). Furthermore, hospitalization for heart failure (HF) was robustly reduced in canagliflozin-treated T2DM patients compared to placebo (HR, 0.67; 95% CI 0.52–0.87) [2] and those with pre-existing HF at baseline derived more benefit [3]. Interestingly, canagliflozin-treated patients were just as likely to suffer an acute myocardial infarction as the placebo group but had a higher chance of surviving such event [2]. These findings support the notion that canagliflozin might have a direct cardiovascular protective effect independently of its antidiabetic action, the mechanism of which is incompletely understood [4, 5].

In recent preclinical studies, it was reported that canagliflozin potently activates adenosine monophosphate (AMP)-activated protein kinase (AMPK) in vitro [6], directly inhibits sodium–hydrogen exchanger (NHE) in non-diabetic healthy hearts [7], and has a direct vasodilatory effect under diabetic conditions [8, 9]. However, it is currently unknown whether acute intravenous administration of canagliflozin has a direct cardiovascular effect independently of antidiabetic action. Accordingly, in the current investigation we sought to characterize the cardiovascular effects of acute intravenous canagliflozin in healthy and infarcted rats.

## Methods

### Animals

Seven-week-old, non-diabetic male Sprague–Dawley rats (body weight [BW]: 250–350 g; Janvier Labs, France) were housed in a room with a constant temperature of 22 ± 2 °C, 12 h light/dark cycles and were allowed access to standard laboratory rat diet and water ad libitum. The investigation conformed to the EU Directive 2010/63/EU and to the *Guide for the Care and Use of Laboratory Animals* published by the US National Institutes of Health (NIH Publication No. 85-23, revised 1996). The experimental protocol was reviewed and approved by the appropriate institutional ethics committee (Reference No. PEI/001/2374-4/2015). The study is interpreted in accordance with the Animal Research: Reporting of In Vivo Experiments (ARRIVE) guidelines [10].

### Materials

Canagliflozin (10 mmol/L) dissolved in dimethyl sulfoxide (DMSO) was obtained from Selleck Chemicals (Munich, Germany). For the in vivo studies, this stock solution of canagliflozin was diluted 1:1000 in 0.9% saline with 1% hydroxyl-propyl-γ-cyclodextrin and was administered intravenously (dose: 3 µg/kg BW). The corresponding vehicle consisted of DMSO diluted 1:1000 in 0.9% saline with 1% hydroxyl-propyl-γ-cyclodextrin. The final intravenous volume of canagliflozin or vehicle bolus was 170–240 µL, varying based on the BW of the given rat (67 µL/100 g BW). For the in vitro study with aortic rings, the stock solution of canagliflozin was used in case of the treatment group, while an equal amount of DMSO was applied in the vehicle/placebo treatment group.

Evans blue (#E2129) and triphenyl-tetrazolium-chloride (TTC; #T8877) were purchased from Sigma Aldrich (Darmstadt, Germany) and used as a 1% solution dissolved in Tris-buffered saline, respectively.

Primary antibodies against phospho-Akt (Ser473, #9271), total-Akt (#9272), phospho-AMPKα (Thr172, #2531), total-AMPKα (#2532), phospho-acetyl-CoA carboxylase (p-ACC; Ser79, #3661), total-ACC (#3662**)**, phospho-eNOS (Ser1177, #9571), total-eNOS (#9572), B-cell leukemia/lymphoma 2 (Bcl-2; #2876), and Bcl-2 associated protein x (Bax; #2772) as well as secondary anti-rabbit horseradish peroxidase-linked antibodies (#7074) were obtained from Cell Signaling Technology (Frankfurt am Main, Germany).

The following TaqMan Gene Expression Assays were purchased from Applied Biosystems (Foster City, CA, USA) and were used for quantitative real-time polymerase chain reaction (PCR): Bax (ID: Rn02532082_g1); Bcl-2 (ID: Rn99999125_m1); catalase (ID: Rn00560930_m1); the 47 kDa subunit of the multiprotein complex nicotinamide adenine dinucleotide phosphate (NADPH) oxidase (p47^phox^; ID: Rn00586945_m1); superoxide dismutase-2 (SOD-2; ID: Rn00690587_g1); ribosomal protein L27 (RPL27; ID: Rn00821099_g1).

For immunohistochemical staining, polyclonal rabbit anti-HNE (4-hydroxynonenal) antibody (#ab46545) was purchased from Abcam, Cambridge, UK. HRP-conjugated secondary antibody (ImmPRESS HRP Reagent Kit, #MP-7401) and black colored nickel–cobalt enhanced diaminobenzidine (#SK4105) were obtained from Vector Laboratories, Burlingame, CA, USA.

For organ bath measurements, phenylephrine (PE), acetylcholine (ACh), sodium nitroprusside (SNP) and potassium chloride (KCl) were purchased from Sigma-Aldrich (Steinheim, Germany) and dissolved in 0.9% sodium chloride (NaCl).

### Study design

In the first part of the in vivo study, non-diabetic rats underwent LAD occlusion for 30 min as detailed below. Vehicle (*IRI*, n = 7; BW = 282 ± 6 g) or canagliflozin (3 µg/kg BW; *IRI *+ *cana*, n = 7; BW = 290 ± 4 g) was administered as a single intravenous bolus at the 5th min of ischemia. After 120 min of reperfusion, serum troponin-T and myocardial infarct size were determined.

In the second part of the in vivo study, non-diabetic rats underwent either sham operation or LAD occlusion for 30 min followed by 120 min reperfusion (IRI) as detailed below. Vehicle or canagliflozin (3 µg/kg BW) was administered as a single intravenous bolus at the 5th min of simulated ischemia (in case of sham operation) or ischemia (in case of LAD occlusion). Accordingly, the following experimental groups participated in the study: *sham *+ *vehicle* (n = 7; BW = 310 ± 10 g); *sham *+ *canagliflozin* (n = 7; BW = 307 ± 11 g); *IRI *+ *vehicle* (n = 9; BW = 308 ± 10 g); *IRI *+ *canagliflozin* (n = 10; BW = 314 ± 7 g). After 120 min of reperfusion, LV function was characterized by pressure–volume (PV) analysis. Then, serum and urine samples were taken to measure markers of hepatic and renal function. Myocardial samples were conserved for immunohistochemical and molecular (protein and mRNA expression) analysis.

In the in vitro study, we preincubated aortic rings of healthy rats (N = 5) with vehicle (DMSO, n = 10 rings) or canagliflozin (10 µM; Canagliflozin, n = 10 rings) to test direct vascular effect of the medication.

### Study protocols

#### Experimental model of in vivo myocardial ischemia–reperfusion injury

Prior to experimentations, rats were allowed to acclimatize. Regional myocardial IRI was induced by transient left anterior descending coronary artery (LAD) ligation in vivo for 30 min, followed by 120 min of reperfusion. Briefly, rats were weighed, anesthesia was induced by i.p. injection of sodium-pentobarbital (60 mg/kg bodyweight) and was maintained by 15–20 mg/kg reinjection when required (by frequently checking pain reflex). The animals were placed in a supine position on a controlled heating pad to maintain the body temperature at 37 ± 0.2 °C throughout the whole protocol. The trachea was cannulated and connected to a small animal respirator. Animals were ventilated with oxygen. Electrocardiographic (ECG) monitoring and serial recording with 6 leads was applied throughout the whole procedure. The left external jugular vein was prepared, and a polyethylene catheter was introduced for intravenous administration of fluid. Thoracotomy was performed at the 4th intercostal space (without cutting ribs), which was spread with a small animal dissector. The pericardium was incised and a 5-0 Prolene suture with a curved needle was placed around the LAD from the right border of the left atrial appendage to the left border of the pulmonary conus, ensuring a universally applicable standard height for LAD ligation in rats [11]. A small pledget was threaded through the ligature and placed in contact with the surface of the myocardium to form a snare. The LAD was occluded for 30 min by placing the ligature through a small piece of plastic tube and pulling the snare tightly in place with a mosquito forceps. Successful occlusion and subsequent development of ischemia was confirmed by (i) prompt ST-segment changes on ECG with progressive ST-segment elevation in at least 3 leads with or without arrythmia, and (ii) decolorization of the myocardium distal to the occlusion. After 30 min of ischemia, the ligature was released and reperfusion was maintained for 120 min. Restoration of blood flow was confirmed by (i) prompt ST-segment changes on ECG with progressive ST-segment normalization/depression and pathological Q-wave formation in leads with previous ST-segment elevation with or without arrythmia, and (ii) re-colorization of the affected myocardium.

Control rats underwent sham operation consisting of the above procedure except the suture around the LAD was not tightened (i.e. no ischemia–reperfusion).

#### Measurement of myocardial infarct size

In the first part of the in vivo study animals were euthanised following 120 min of reperfusion. The abdominal aorta of the animals was cannulated, arterial blood samples were collected for serum troponin-T measurement. Infusion of 4 °C ringer solution (50 mL) was then performed retrogradely. Then, the suture was retightened around the LAD and 1% Evans blue was retrogradely injected into the aorta to delineate the area at risk (AAR). Hearts were harvested and allowed to freeze at − 20 °C before being sliced into 2 mm thick transverse sections in a heart matrix (Zivic Instruments; Pittsburgh, USA), after which they were incubated in 37 °C 1% triphenyl-tetrazolium-chloride (TTC) for 15 min. Intracellular dehydrogenases in the myocardium react with TTC, which results in the red staining of the viable area, while the infarcted (non-viable) zones remain white. The stained heart slices were digitally scanned and evaluated with software planimetry by an experienced investigator blinded to the experimental design of the study. The area at risk was quantified as the percentage of the total ventricular area, while infarct size (IS) was expressed as the percentage of the AAR.

#### Left ventricular pressure–volume analysis

In the second part of the in vivo study, PV analysis was performed as described previously [12], with a microtip pressure-conductance catheter (SPR-838; Millar Instruments, Houston, TX, USA) inserted into the LV via the right common carotid artery. For analysis, all PV loops were obtained with the ventilator turned off for 5–10 s and the animal apnoeic.

Heart rate, end-systolic blood pressure, maximal slope of systolic pressure increment (dP/dt_max_), arterial elastance, the slope of the end-diastolic PV relationship (EDPVR) and time constant of LV pressure decay (Tau_Weiss_) were calculated with PV analysis software (PVAN; Millar Instruments, Houston, TX, USA). Rate-pressure product (RPP, or double product) was calculated as heart rate multiplied by LV systolic pressure.

To detect load-independent sensitive contractility parameters, PV loops were also registered during transiently decreasing preload achieved by the transient occlusion of the inferior vena cava. Accordingly, the slope of the end-systolic PV relationship (ESPVR) and preload recruitable stroke work (PRSW) were calculated. To assess LV mechanoenergetics, ventriculo-arterial coupling (VAC) was calculated as the ratio of arterial elastance and ESPVR, and mechanical efficiency was computed. After PV measurements, animals were euthanized, the abdominal aorta was cannulated, arterial blood samples were collected and 4 °C ringer solution (50 mL) was infused retrogradely. Hearts were then harvested, and a cross section of the hearts (at the level of the ventricles) was placed in 4% buffered paraformaldehyde for the forthcoming immunohistochemical analysis, while LV samples taken from the AAR were immediately snap frozen in liquid nitrogen for the forthcoming immunoblot and PCR measurements.

#### Western blot analysis

Left ventricular myocardial tissue samples from the AAR were lysed mechanically by the Tissue Lyzer LT system (Qiagen; Hilden, Germany) and chemically by RIPA buffer (Melford, Ipswich, UK) containing protease and phosphatase inhibitor cocktail (Roche; Mannheim, Germany). The concentrations of the extracted proteins were measured by Bradford assay. Then, protein homogenates were suspended in sample buffer and boiled at 95 °C for 5 min. A total of 40 µg protein for each sample was loaded onto 6–12% acrylamide gels and separated with sodium dodecyl sulfate polyacrylamide gel-electrophoresis (Peqlab Biotechnologie; Erlangen, Germany). Gels were transferred to polyvinylidene fluoride membranes (Millipore; Darmstadt, Germany) under semi-dry conditions. Membranes were then washed and blocked for 1 h in 5% bovine serum albumin (BSA) in Tris-buffered saline Tween 20 (TBST) at room temperature. The membranes were incubated overnight at 4 °C with the following primary antibodies (1:1000, 2.5% BSA in TBST): phospho-Akt (Ser473), total-Akt, phospho-AMPKα (Thr172), total-AMPKα, phospho-ACC (Ser79), total-ACC, phospho-eNOS (Ser1177), total-eNOS, Bax and Bcl-2, respectively. The blots were washed and incubated with horseradish peroxidase- conjugated secondary antibody (1:5000, 2.5% BSA in TBST) for 1 h at room temperature. The immunoreactive protein bands were developed using the Enhanced Chemiluminescence system (PerkinElmer; Rodgau-Juegesheim, Germany). The intensity of the immunoblot bands was analyzed with Chemi-smart 5100 (Peqlab Biotechnologie).

#### Quantitative real-time polymerase chain reaction

Left ventricular myocardial tissue samples from the AAR were homogenized, total RNA was isolated by using RNeasy Fibrous Tissue Kit (Qiagen; Hilden, Germany) according to the manufacturer’s instructions. RNA concentration was measured photometrically at 260 nm, RNA purity was ensured by obtaining a 260/280 nm optical density ratio of ∼ 2.0 and RNA was reverse transcribed to cDNA with QuantiTect Reverse Transcription Kit (Qiagen) by using 1 μg RNA of each sample and random primers. After that, qRT-PCR was performed on a StepOnePlus RT PCR System (Applied Biosystems; Foster City, CA, USA) using TaqMan Universal PCR MasterMix and TaqMan Gene Expression Assays (Applied Biosystems). Every sample was quantified in triplicates in a volume of 10 μL in each well containing cDNA (1 μL). The following targets were investigated: Bax; Bcl2; catalase; SOD-2; p47^phox^. Data were normalized to the housekeeping RPL27. Gene expression levels were calculated using the comparative method (2^−ΔCT^).

#### Immunohistochemistry

Heart samples of the harvested hearts were fixed in 4% buffered paraformaldehyde for 24 h, embedded in paraffin and 5 µm thick sections were cut.

In order to asses myocardial oxidative stress, staining for HNE was performed. After deparaffinization and antigen retrieval (0.1 mmol/L citrate buffer, pH = 3, heating in microwave oven for 15 min) sections were incubated with polyclonal rabbit anti-HNE antibody (1:500, overnight, 4 °C). HRP-conjugated secondary antibody (30 min, room temperature) and black colored nickel–cobalt enhanced diaminobenzidine (7 min, room temperature) were used to visualize the labeling. Light microscopic examination was performed using a Nikon Eclipse Ni Microscope (Nikon Instruments, Amstelveen, Netherlands) and a digital image of the LV area of interest was captured in each section (from each animal) using Nikon DS-RI2 camera (Nikon Instruments) and imaging software at 100× magnification. Staining intensity was determined using ImageJ Software (National Institutes of Health, Bethesda, MD, USA). The percentage of positively stained tissue area to total LV area of each image was calculated. Immunohistochemical evaluation was performed by a person blinded to the study groups.

#### In vitro organ bath experiments

Sections of the thoracic aortas were harvested from healthy rats as described previously [13], and were incubated in either DMSO or 10 µM canagliflozin (a clinically relevant plasma concentration [14]) for 30 min, respectively (for each group: n = 10 aortic rings). After washing and equilibration, rings were pre-constricted with PE (10^−6^ M), and relaxation responses were examined by adding cumulative concentrations of the endothelium-dependent vasodilator ACh (10^−9^–10^−4^ M). Following PE-induced re-constriction, cumulative concentrations of the endothelium-independent vasodilator SNP (10^−10^–10^−5^ M) were added. Half-maximal response (EC_50_) values were obtained from individual concentration–response by fitting experimental data to a sigmoidal equation. The sensitivity to vasodilators was assessed by pD_2_ = − logEC_50_ (M), vasorelaxation (and its maximum (*R*_max_) is expressed as percentage of the contraction induced by PE.

#### Serum and urine measurements

Serum and urine samples were collected from animals after the protocol, and markers of hepatic and renal function as well as serum troponin-T were determined in the Central Laboratory of Heidelberg University Hospital (Heidelberg, Germany). Serum troponin-T levels were analysed using the Troponin T hs STAT Reagent Kit (#05092728; Roche Diagnostics Gmbh, Mannheim, Germany) according to the manufacturer’s protocol.

### Statistical analysis

All values are expressed as mean ± SEM. Statistical analysis was performed with GraphPad Prism 7 (GraphPad Software Inc., San Diego, CA, USA). Normal distribution of the data was evaluated by Shapiro–Wilk test. Significance of differences between two groups was assessed using unpaired two-sided Student *t*-test. In case of four groups, two-way analysis of variance (ANOVA) was performed with the factors ‘IRI’ (ischemia–reperfusion injury; P_IRI_) and ‘CANA’ (canagliflozin treatment; P_CANA_), and their interaction (P_int_) was evaluated. In case of two-way ANOVA, Tukey’s post hoc test was applied to examine intergroup differences. A value of P < 0.05 was considered statistically significant.

## Results

### Acute canagliflozin treatment decreased myocardial infarct size

**Fig. 1:**
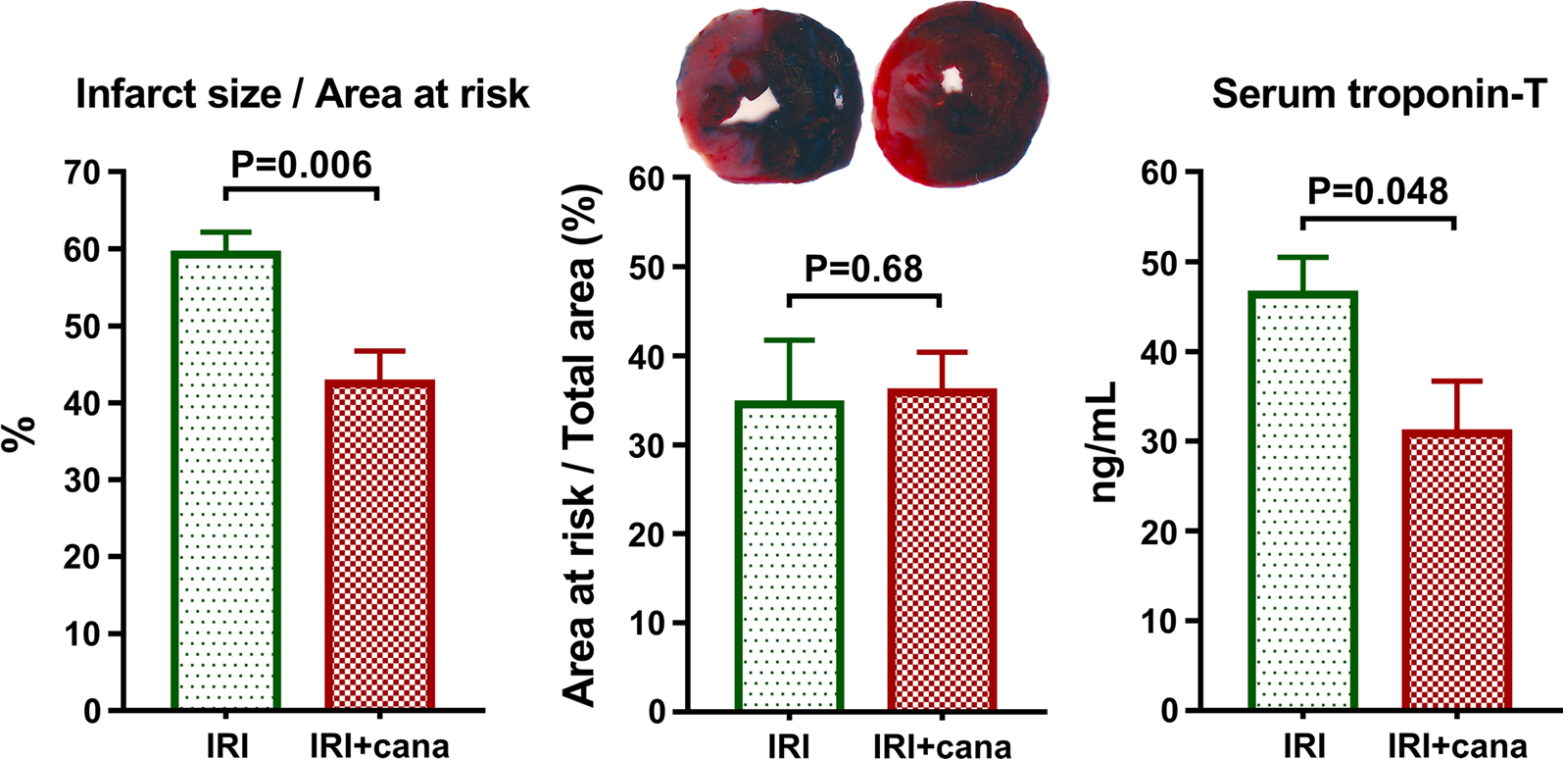
Measurement of myocardial infarct size and serum troponin-T. Myocardial infarct size of vehicle-treated (*IRI*, n = 7) and canagliflozin-treated (*IRI *+ *cana*, n = 7) rats with myocardial infarction is depicted as percentage of area at risk. Area at risk (the area distal to the occlusion) is expressed as the percentage of the total ventricular area in each group. Serum troponin-T values of these groups are depicted. Student *t*-test P values are reported with ticked lines between two groups. *cana* canagliflozin, *IRI* ischemia–reperfusion injury

A single intravenous bolus of canagliflozin 5 min after the onset of ischemia resulted in a ~ 30% IS reduction compared to vehicle (42.5 ± 2.9% vs. 59.3 ± 4.2%, P = 0.006), while the size of the area distal to the occlusion (i.e. AAR) did not differ (Fig. 1). Canagliflozin significantly (P = 0.048) reduced the myocardial necrosis marker serum troponin-T level (Fig. 1).

### Acute canagliflozin treatment alleviated left ventricular systolic and diastolic dysfunction following ischemia–reperfusion injury

**Fig. 2:**
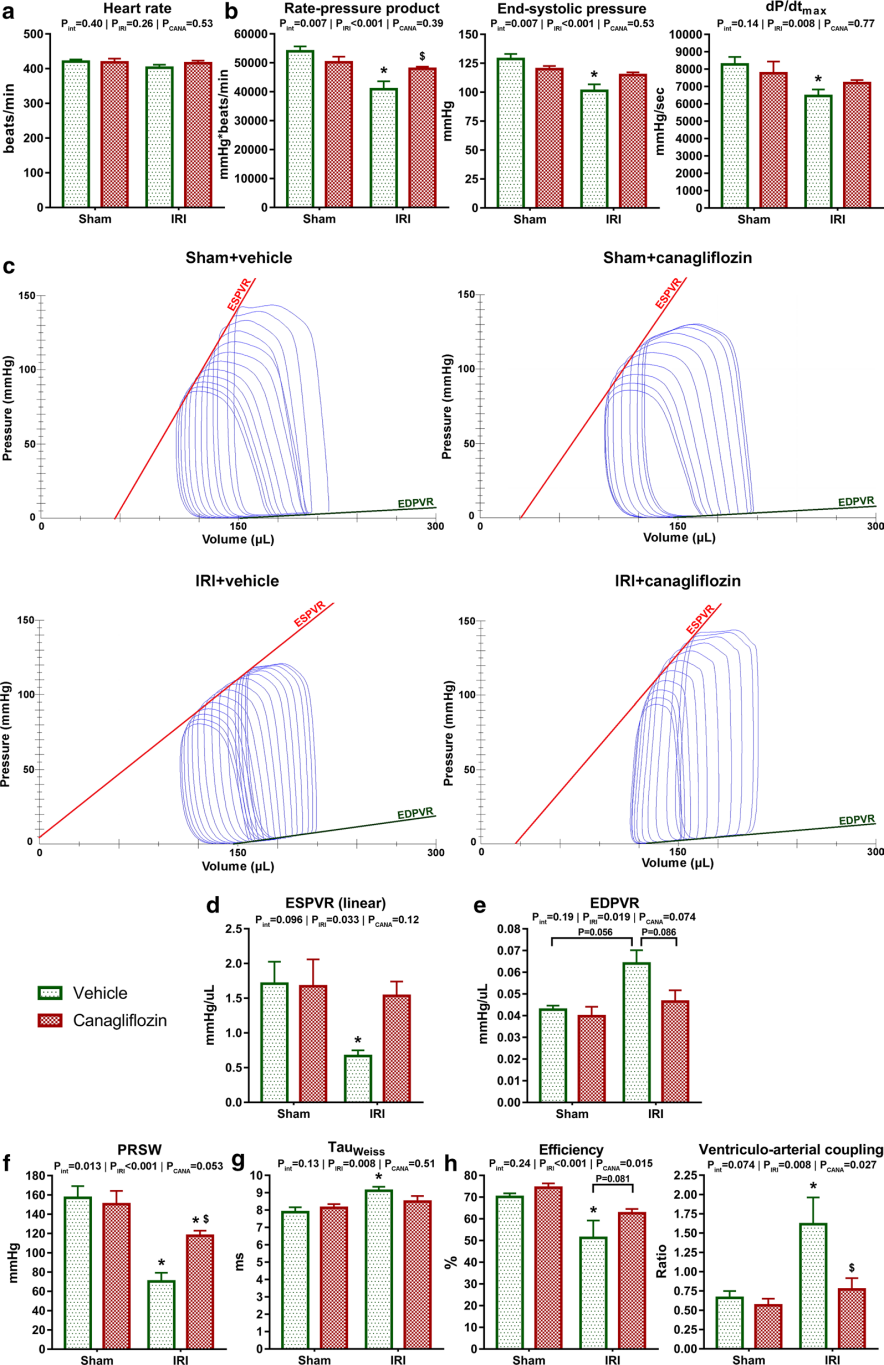
In vivo left ventricular (LV) pressure–volume (PV) analysis. **a** Heart rate values. **b** Preload-dependent LV contractility indices. **c** Representative PV loops from one representative animal of each group recorded during the transient occlusion of the inferior vena cava. **d**, **f** Preload-independent LV contractility indices. **e**, **g** Markers of LV diastolic function. **h** Indices of LV mechanoenergetics. The number of rats in each experimental group: *sham *+ *vehicle* (n = 7); *sham *+ *canagliflozin* (n = 7); *IRI *+ *vehicle* (n = 9); *IRI *+ *canagliflozin* (n = 10). Two-way analysis of variance (ANOVA) P values (with factors: ischemia–reperfusion injury [P_IRI_] and canagliflozin treatment [P_CANA_]; and their interaction [P_int_]) are depicted under the title of each graph for the given variable. Tukey’s post hoc P values are reported with ticked lines between two groups or as follows: *P < 0.05 versus *sham *+ *vehicle*; ^$^P < 0.05 versus *IRI *+ *vehicle*. *dP/dt*_*max*_ maximal slope of systolic pressure increment; *EDPVR* end-diastolic PV relationship, *ESPVR* slope of the end-systolic PV relationship, *IRI* ischemia–reperfusion injury, *Tau*_*Weiss*_ time constant of LV pressure decay

Without affecting heart rate (Fig. 2a), canagliflozin promoted LV functional recovery following IRI reflected by significantly (P = 0.045) increased RPP compared with vehicle, and effectively alleviated systolic dysfunction shown by preserved LV end-systolic pressure and dP/dt_max_ (Fig. 2b). In case of LV end-systolic pressure and RPP, there was a significant interaction (P_int_ = 0.007, respectively) between the factors of IRI and canagliflozin treatment, suggesting a disease-specific effect of the medication (Fig. 2b). Because the latter parameters are dependent on pre- and afterload, we recorded load-independent, sensitive contractility indices. In vehicle-treated MI rats, we observed a significant (P < 0.05, respectively) reduction in the value of ESPVR (Fig. 2d) and PRSW (Fig. 2f) compared with sham operated littermates. On the contrary, canagliflozin kept ESPVR values on a level comparable to that of controls. Furthermore, canagliflozin maintained a significantly (P = 0.010) higher PRSW compared to vehicle-treated MI rats, which was a disease-specific effect (P_int_ = 0.013) (Fig. 2f).

Tau_Weiss_, which is a sensitive index of active relaxation, was significantly (P = 0.027) prolonged in vehicle-treated MI rats, reflecting a compromised LV diastolic function (Fig. 2g). On the contrary, canagliflozin treatment prevented the prolongation of Tau_Weiss_. The value of EDPVR (stiffness marker) was increased (P = 0.057) in vehicle-treated infarcted rats but remained comparable to that of controls with canagliflozin treatment (Fig. 2e).

VAC is a ratio that represents the coupling between LV contractility and arterial afterload. VAC was found to be significantly (P = 0.018) impaired in vehicle-treated MI rats but was substantially (P = 0.022) alleviated by canagliflozin treatment (Fig. 2h). Consistently, LV mechanical efficiency was optimized by canagliflozin treatment following IRI (Fig. 2h).

### Acute canagliflozin treatment modulated phosphorylation of cardioprotective signalling mediators

**Fig. 3:**
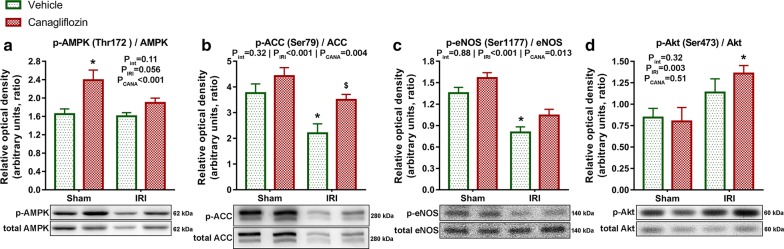
Effect of canagliflozin treatment on the phosphorylation of AMPK, ACC, eNOS and Akt. **a** Representative blots and quantification of phosphorylation of AMPK at the Thr172 activation site normalized to total AMPK expression. **b** Representative blots and quantification of phosphorylation of ACC at the AMPK specific Ser79 residue normalized to total ACC expression. **c** Representative blots and quantification of phosphorylation of eNOS at the Ser1177 residue normalized to total eNOS expression. **d** Representative blots and quantification of phosphorylation of Akt at the Ser473 residue normalized to total Akt expression. The number of rats in each experimental group: *sham *+ *vehicle* (n = 7); *sham *+ *canagliflozin* (n = 7); *IRI *+ *vehicle* (n = 9); *IRI *+ *canagliflozin* (n = 10). Two-way analysis of variance (ANOVA) P values (with factors: ischemia–reperfusion injury [P_IRI_] and canagliflozin treatment [P_CANA_]; and their interaction [P_int_]) are depicted under the title of each graph for the given variable. Tukey’s post hoc P values are reported as follows: *P < 0.05 versus *sham *+ *vehicle*; ^$^P < 0.05 versus *IRI *+ *vehicle*. *ACC* acetyl-CoA carboxylase, *AMPK* adenosine monophosphate (AMP)-activated protein kinase, *eNOS* endothelial nitric-oxide synthase

A single intravenous bolus of canagliflozin had a major (P_CANA_ < 0.001) impact on phosphorylation of AMPK at the Thr172 activation site compared with vehicle treatment, which effect was not disease-specific (P_int_ = 0.11) (Fig. 3a). Accordingly, canagliflozin had a significant (P_CANA_ = 0.004) impact on the phosphorylation of the downstream ACC at the AMPK specific Ser79 residue, with significant (P = 0.025) difference between canagliflozin-treated versus vehicle-treated MI rats (Fig. 3b), suggesting an increased activation of AMPK in infarcted rats. Phosphorylation of eNOS at the Ser1177 residue is crucial for nitric-oxide (NO) production [15]. Canagliflozin treatment had a significant (P_CANA_ = 0.013) impact on eNOS phosphorylation, which was not disease-specific (P_int_ = 0.88) (Fig. 3c). Myocardial IRI had a significant (P_IRI_ < 0.001) negative effect on eNOS phosphorylation, which was substantially (P < 0.001) lower in the vehicle-treated MI group compared to that of sham-operated rats (Fig. 3c). However, canagliflozin prevented IRI-induced reduction of eNOS phosphorylation (Fig. 3c), reflecting a maintained NO production.

In canagliflozin-treated MI rats, the phosphorylation of Akt (a well-known cardioprotective mediator in the setting of acute myocardial IRI) was significantly (P = 0.024) higher compared to sham-operated rats (Fig. 3d). Notably, canagliflozin did not increase phosphorylation of Akt in healthy rats (Fig. 3d).

### Acute canagliflozin treatment reduced expression of apoptotic and nitro-oxidative stress markers, and reduced myocardial 4-hydroxynonenal positivity

**Fig. 4:**
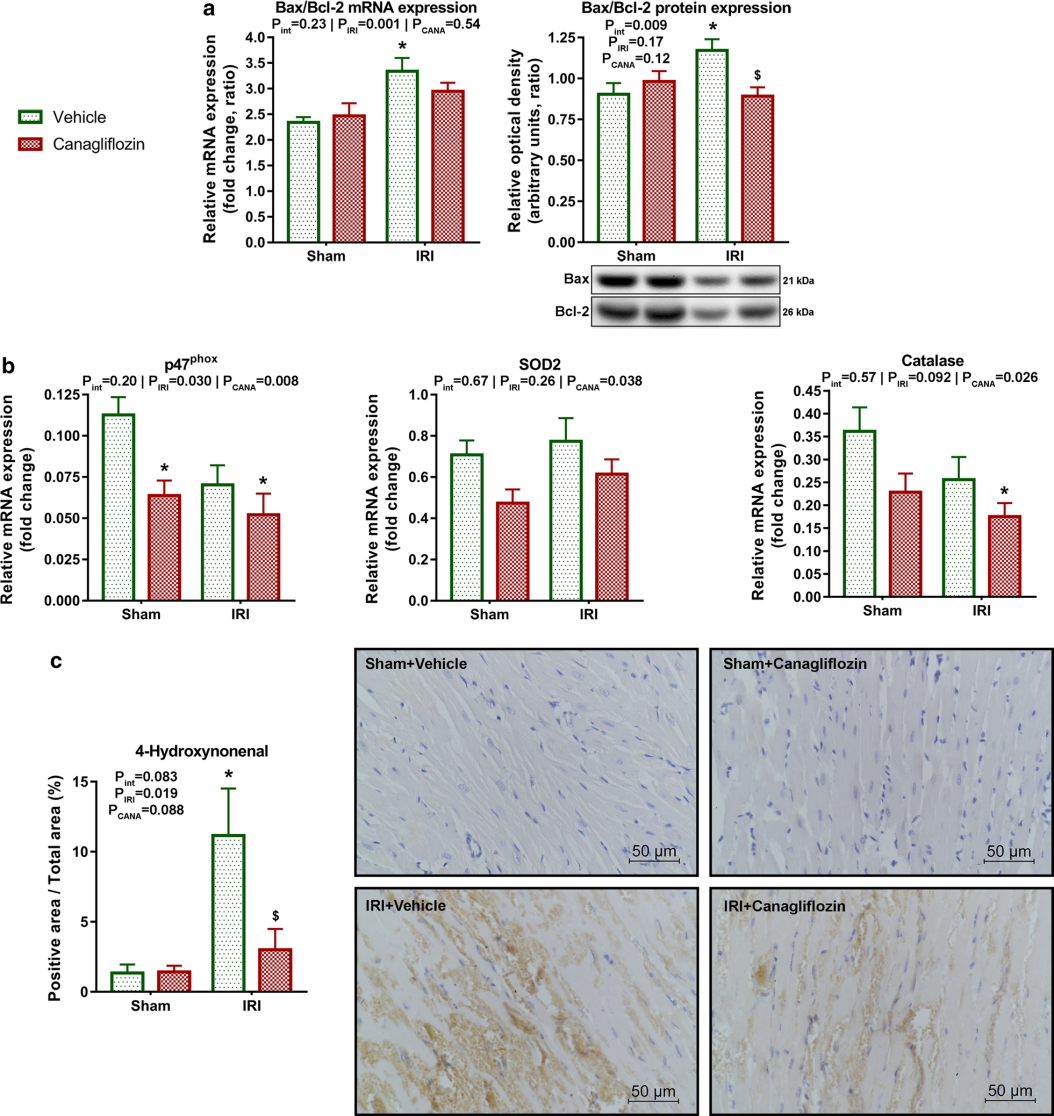
Effect of canagliflozin treatment on apoptotic and oxidative stress markers. **a** mRNA expression of Bax normalized to that of Bcl-2. Protein expression analysis depicts representative blots and quantification of Bax expression normalized to that of Bcl-2. **b** mRNA expression analysis of p47^phox^, SOD2 and catalase normalized to the housekeeping RPL27, respectively. **c** Quantification and representative sections of 4-hydroxynonenal (HNE) staining of the LV. The number of rats in each experimental group: *sham *+ *vehicle* (n = 7); *sham *+ *canagliflozin* (n = 7); *IRI *+ *vehicle* (n = 9); *IRI *+ *canagliflozin* (n = 10). Two-way analysis of variance (ANOVA) P values (with factors: ischemia–reperfusion injury [P_IRI_] and canagliflozin treatment [P_CANA_]; and their interaction [P_int_]) are depicted under the title of each graph for the given variable. Tukey’s post hoc P values are reported as follows: *P < 0.05 versus *sham *+ *vehicle*; ^$^P < 0.05 versus *IRI *+ *vehicle*. Bax = B-cell leukemia/lymphoma 2 associated protein x; *Bcl2* B-cell leukemia/lymphoma 2, *p47*^*phox*^ the 47 kDa subunit of the multiprotein complex nicotinamide adenine dinucleotide phosphate (NADPH) oxidase, *SOD2* superoxide dismutase-2

The ratio of the relative mRNA expression of the pro-apoptotic Bax and anti-apoptotic Bcl-2 was significantly (P = 0.011) elevated in the vehicle-treated MI group compared with the sham-operated group (Fig. 4a). In MI rats, canagliflozin treatment kept Bax/Bcl-2 mRNA expression levels comparable to that of controls (Fig. 4a). Protein expression analysis of the Bax/Bcl-2 ratio showed a significant (P = 0.039) increase in non-treated MI hearts, which was significantly (P = 0.014) reduced by canagliflozin treatment (Fig. 4a). This was a disease-specific effect (P_int_ = 0.009).

Canagliflozin treatment reduced (P_CANA_ < 0.05, respectively) the mRNA expression of genes related to nitro-oxidative stress: p47^phox^, SOD2, and catalase (Fig. 4b), which was not a disease-specific effect. Furthermore, vehicle-treated infarcted hearts had a significantly (P = 0.027) higher level of HNE positivity compared to sham-operated controls. On the contrary, canagliflozin treatment in the infarcted group significantly (P = 0.037) lowered the level of LV HNE positivity compared to vehicle treatment (Fig. 4c).

### Preincubation with canagliflozin promoted endothelium-dependent vasorelaxation in aortic rings of healthy rats

**Fig. 5:**
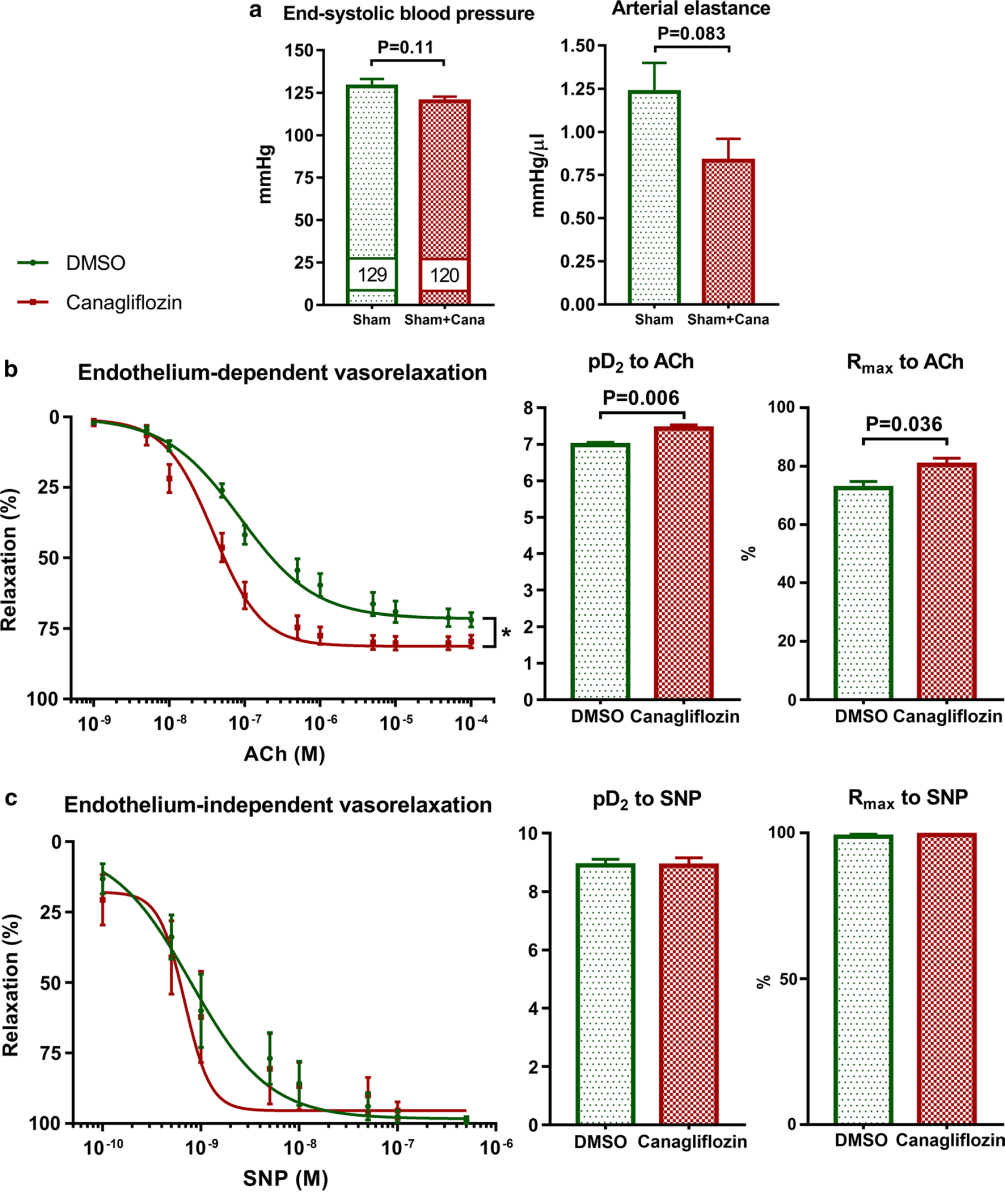
Effect of canagliflozin on vasorelaxation. **a** End-systolic blood pressure and arterial elastance of vehicle-treated (*sham *+ *vehicle*, n = 7) and canagliflozin-treated (*sham *+ *canagliflozin*, n = 7) healthy (sham-operated) rats from the second part of the in vivo study. **b** Effect of canagliflozin on endothelium-dependent vasorelaxation in healthy aortic rings in vitro (n = 10 aortic rings in each group). **c** Effect of canagliflozin on endothelium-independent vasorelaxation in healthy aortic rings in vitro (n = 10 aortic rings in each group). Student *t*-test P values are reported with ticked lines between two groups or as: *P < 0.05. *ACh* acetylcholine, *cana* canagliflozin, *DMSO* dimethyl sulfoxide, *pD*_*2*_ − logEC_50_, *R*_*max*_ maximal vasorelaxation, *SNP* sodium nitroprusside

During LV functional PV analysis, tendential reduction in LV end-systolic pressure and arterial elastance was found in canagliflozin-treated sham-operated (*sham *+ *canagliflozin*) rats compared to vehicle-treated sham-operated (*sham *+ *vehicle*) littermates (Fig. 5a). In order to investigate whether this afterload reduction derived from a direct vasoactive property of the drug, we carried out organ bath measurements with healthy aortic rings. Compared to vehicle (DMSO), canagliflozin preincubation significantly (P = 0.006) increased sensitivity of aortic rings to ACh-induced vasorelaxation (pD_2_ to ACh) as well as the maximal vasorelaxation (R_max_ to ACh, P = 0.036) (Fig. 5b), indicating an enhanced endothelium-dependent nitric-oxide (NO) mediated vasorelaxation. Canagliflozin had no effect on endothelium-independent vasodilation (Fig. 5c).

### Acute canagliflozin treatment did not alter blood and urine glucose levels and had no adverse effect on hepatic and renal function

Canagliflozin had no effect on blood and urinary glucose in normoglycemic rats (Table 1). Treatment was not associated with hepatic or renal injury reflected by the serum and urinary markers (Table 1).

**Table 1:** Serum and urine parameters

*Parameters*	Sham + vehicle	Sham + canagliflozin	IRI + vehicle	IRI + canagliflozin
Serum glucose (mg/dL)	215 ± 15	193 ± 10	205 ± 13	226 ± 17
Urine glucose (mmol/L)	0.54 ± 0.06	0.49 ± 0.06	0.44 ± 0.06	0.52 ± 0.04
Serum creatine kinase (IU/L)	1778 ± 247	1806 ± 346	3445 ± 436*	3089 ± 477
Serum lactate dehydrogenase (IU/L)	961 ± 81	789 ± 41	3279 ± 259*	2716 ± 242*
Serum aspartate transaminase (IU/L)	217 ± 23	191 ± 10	899 ± 119*	745 ± 91*
Serum alanine transaminase (IU/L)	63 ± 4	57 ± 5	131 ± 11*	114 ± 12*
Alkaline phosphatase (IU/L)	268 ± 10	277 ± 13	273 ± 13	304 ± 15
Cholesterol (mg/dL)	79 ± 4	82 ± 4	78 ± 4	77 ± 2
Creatinine (mg/dL)	0.47 ± 0.05	0.42 ± 0.02	0.58 ± 0.08	0.55 ± 0.05
Urinary urea nitrogen (g/dL)	27 ± 5	26 ± 3	21 ± 4	20 ± 2
Urinary creatinine (mg/dL)	103 ± 9	115 ± 11	116 ± 12	99 ± 8
Urinary albumin (mg/L)	16 ± 3	13 ± 6	16 ± 4	15 ± 3

Canagliflozin did not alter serum and urine glucose levels in non-diabetic rats, and was not associated with hepatic or renal injury

To investigate intergroup differences, Tukey’s post hoc test was performed following two-way analysis of variance (ANOVA). *P < 0.05 versus *sham *+ *vehicle*; *IRI* ischemia–reperfusion injury

## Discussion

For the first time, we demonstrated that the intravenous administration of the SGLT2 inhibitor canagliflozin after the onset of ischemia protected against in vivo myocardial IRI in non-diabetic rats. Canagliflozin increased the phosphorylation of cardioprotective signalling mediators while reducing the expression of apoptotic and nitro-oxidative stress markers. Accordingly, canagliflozin prevented the development of systolic and diastolic dysfunction following IRI. The medication had a slight blood pressure and LV afterload lowering effect in healthy rats, and enhanced endothelium-dependent vasorelaxation in aortic rings.

The main antidiabetic action of SGLT2 inhibitors in T2DM derives from the blockade of glucose reabsorption in the proximal tubule of the kidney. According to Zelniker and Braunwald [5], SGLT2 inhibition in the kidney does not serve as full explanation for the stunning cardiovascular protection afforded by SGLT2 inhibitors in clinical trials, yet other effects than their antihyperglycemic actions are poorly understood. The CANVAS Program clinical trial showed fast separation of event curves in canagliflozin-treated T2DM patients compared to placebo, and outstanding reduction in hospitalization for HF [2] (especially in those with HF at baseline [3]), that was also documented in a real-world study (CVD-REAL) [16]. These findings suggest that the cardioprotective effect of canagliflozin might be partly independent of its antidiabetic action. This is of particular interest, since the mRNA expression of SGLT2 in the human heart is negligible [17], suggesting potential off-target effects of the medication.

In the present study we found that a single intravenous bolus of canagliflozin affected AMPK activation independently of antidiabetic action in rats with healthy and infarcted hearts. This finding contributes to previous preclinical experiments which have shown that canagliflozin potently and immediately activates AMPK in cancer cells [18], murine hepatocytes [6], and human endothelial cells [19]. Studies have shown that AMPK activation has beneficial effects in various cardiovascular diseases [20]. Specifically, pharmacological AMPK activation has been documented to protect against myocardial IRI in non-diabetic [21] and diabetic [22] mice, which was mediated by increased eNOS phosphorylation. Here we present that canagliflozin increased the activation of AMPK given after the onset of ischemia (in a clinically relevant fashion), as evidenced by a significant upregulation of the phosphorylation of ACC at the AMPK specific site. The medication prevented the reduction of eNOS phosphorylation following IRI compared to vehicle, suggesting a preserved NO signaling. Maintained NO signaling has been previously shown to reduce apoptosis, myocardial nitro-oxidative stress and platelet aggregation, conferring protection against myocardial IRI [23]. Akt (also referred to as protein kinase B) has also been implicated as a crucial mediator of cardioprotection during myocardial IRI, and has been demonstrated to phosphorylate eNOS at the same site (Ser1177) as AMPK [24]. We found a significantly increased Akt phosphorylation in canagliflozin-treated infarcted hearts compared to sham-operated controls. It is not clear from our study as to what extent AMPK or Akt activation contributed to the preserved phosphorylation of eNOS in the infarcted hearts, and further studies are warranted to elucidate this. Both AMPK [25] and Akt [26] have been previously implicated in reducing cell death during myocardial ischemia–reperfusion. In the present study, canagliflozin decreased the ratio of Bax/Bcl-2 expression on the mRNA and protein levels following myocardial IRI, suggesting reduced apoptotic activity in the AAR compared to vehicle treatment. Accordingly, we report a ~ 30% reduction in infarct size and serum troponin-T levels in canagliflozin-treated infarcted rats compared to vehicle-treated ones.

Oxidative stress is a key mediator of myocardial IRI as it contributes to cell death and affects myocardial infarct size [27]. In our study, canagliflozin treatment reduced the mRNA expression of p47^phox^ (subunit of the pro-oxidative NADPH oxidase), SOD2 and catalase in the myocardium, which is in good agreement with a previous study in which pharmacological activation of AMPK downregulated the expression of these enzymes following renal IRI [28]. To further elucidate the possible antioxidative effect of canagliflozin, we performed immunohistochemical staining for HNE. The burst of reactive oxygen species during myocardial IRI results in lipid peroxidation, during which the most toxic aldehydic end-product, HNE, accumulates. In the myocardium, evidence shows that HNE disrupts enzymatic and mitochondrial functions [29]. Hence, the level of HNE production has been shown to correlate with the extent of myocardial IRI in terms of functional recovery [30]. In the present study, we documented a significantly lower HNE positivity in the canagliflozin-treated infarcted hearts compared to vehicle-treated ones. This might reflect that canagliflozin reduced myocardial oxidative stress, contributing to an enhanced LV functional recovery following myocardial IRI.

The present study provides a detailed functional analysis of healthy and infarcted rat hearts, treated with either vehicle or canagliflozin. We show that a single bolus of intravenous canagliflozin given in a clinically relevant approach (i.e. after the onset of ischemia) substantially improved recovery of the myocardium following IRI, as evidenced by significantly higher RPP and increased LV end-systolic pressure and dP/dt_max_. These are in accordance with a previous study, in which a pharmacological AMPK activator increased RPP following IRI, indicating an improved LV recovery [21]. The values of ESPVR and PRSW, which are load-independent sensitive contractility indices, showed a marked amelioration in canagliflozin-treated MI rats compared to vehicle-treated ones. These findings reflect the protective effect of canagliflozin against the development of LV systolic dysfunction. Accordingly, we found a significantly improved VAC in canagliflozin-treated infarcted hearts compared to vehicle-treated ones, suggesting a more physiological matching between LV contractility and arterial afterload. Regarding diastolic function, we found a severely compromised LV stiffness (EDPVR) and active relaxation (Tau_Weiss_) in rats with acute MI. However, canagliflozin normalized EDPVR and prevented the prolongation of the time-constant of active relaxation (Tau_Weiss_), suggesting a preserved LV diastolic function following IRI. Finally, the severe attenuation of LV mechanical efficiency in vehicle-treated infarcted hearts was prevented by canagliflozin treatment, indicating decreased oxygen consumption of the myocardium to maintain cardiac output.

In healthy non-diabetic rats, we show that acute canagliflozin treatment slightly decreased systolic blood pressure and the afterload index arterial elastance without inducing reflex tachycardia. Canagliflozin treatment reduced systolic blood pressure in T2DM patients in the clinical setting at very early stages [2, 31]. In previous preclinical studies, a possible effect of canagliflozin on vasorelaxation was assessed. Canagliflozin incubation was found to induce vasorelaxation in pulmonary but not coronary artery rings from diabetic mice [8], and increased vasorelaxation response to ACh in hyperglycemic aortic rings [9]. These findings suggest a direct vascular effect of canagliflozin that is not related to antihyperglycemic effect. Indeed, in ex vivo non-diabetic mouse hearts, canagliflozin preincubation significantly reduced perfusion pressure, suggesting enhanced coronary vasodilation [7]. Further contributing to these findings, here we report that preincubation of aortic rings of non-diabetic rats with a clinically relevant concentration of canagliflozin enhanced vasorelaxation sensitivity to ACh and increased overall maximal relaxation of the rings, indicating that canagliflozin might have a direct vasorelaxant effect independently of its antidiabetic action. This might explain why a tendential reduction in cardiac afterload was seen in healthy treated rats in vivo.

Our study has limitations. First, we cannot rule out the influence of cardioprotective molecular mechanisms other than those investigated in the present work, which could have contributed to the cardioprotection exerted by canagliflozin. Accordingly, further studies are required to ascertain the involvement of such pathways. Second, we tested the medication only in male non-diabetic rats. Additional experiments are warranted to characterize possible sex-specific differences regarding the cardioprotective effect of canagliflozin and to investigate whether it protects against myocardial IRI in diabetic rats.

## Conclusions

The impressive cardiovascular protective effects of canagliflozin in T2DM patients reported in clinical trials seem to be partly independent of its antihyperglycemic action, because canagliflozin affects cardioprotective signalling in non-diabetic rats, and enhances endothelium-dependent vasorelaxation. Accordingly, canagliflozin protected against myocardial IRI and alleviated systolic and diastolic dysfunction.

## Abbreviations

AAR: area at risk
ACC: acetyl-CoA carboxylase
ACh: acetylcholine
AMPK: adenosine monophosphate-activated protein kinase
ANOVA: analysis of variance
ARRIVE: Animal Research: Reporting of In Vivo Experiments
Bax: B cell leukemia/lymphoma-2 associated protein x
Bcl-2: B-cell leukemia/lymphoma-2
BSA: bovine serum albumin
CANA: canagliflozin
DMSO: dimethyl sulfoxide
ECG: electrocardiography/electrocardiographic
EDPVR: end-diastolic pressure–volume relationship
eNOS: endothelial nitric-oxide synthase
ESPVR: end-systolic pressure–volume relationship
HF: heart failure
IRI: ischemia–reperfusion injury
IS: infarct size
KCl: potassium chloride
LAD: left anterior descending coronary artery
LV: left ventricle/left ventricular
MI: myocardial infarction
NaCl: sodium chloride
p47^phox^: 47 kDa subunit of the multiprotein complex nicotinamide adenine dinucleotide phosphate (NADPH) oxidase
pD_2_: sensitivity to vasodilators (pD_2_: − logEC_50_)
PE: phenylephrine
PV: pressure–volume
PRSW: preload recruitable stroke work
R_max_: maximal vasorelaxation
RPL27: ribosomal protein L27
RPP: rate-pressure product (also referred to as double product)
SGLT2: sodium–glucose cotransporter-2
SNP: sodium nitroprusside
SOD2: superoxide dismutase-2 (also referred to as manganese superoxide dismutase)
T2DM: type 2 diabetes mellitus
Tau_Weiss_: time constant of left ventricular pressure decay
TBST: Tris-buffered saline Tween 20
TTC: triphenyl-tetrazolium-chloride
VAC: ventriculo-arterial coupling
